# Lumbar segment-dependent soft tissue artifacts of skin markers during *in vivo* weight-bearing forward–Backward bending

**DOI:** 10.3389/fbioe.2022.960063

**Published:** 2022-08-17

**Authors:** Xin Xi, Zhi Ling, Cong Wang, Chunya Gu, Xuqiang Zhan, Haixin Yu, Siqi Lu, Tsung-Yuan Tsai, Yan Yu, Liming Cheng

**Affiliations:** ^1^ Department of Spine Surgery, Tongji Hospital, School of Medicine, Tongji University, Shanghai, China; ^2^ Key Laboratory of Spine and Spinal Cord Injury Repair and Regeneration, Ministry of Education, Department of Spine Surgery, Tongji Hospital, School of Medicine, Tongji University, Shanghai, China; ^3^ School of Biomedical Engineering, Shanghai Jiao Tong University, Shanghai, China; ^4^ Department of Spinal Rehabilitation, Tongji Hospital, School of Medicine, Tongji University, Shanghai, China; ^5^ Department of Orthopedic Surgery, Yangpu Hospital, School of Medicine, Tongji University, Shanghai, China; ^6^ TAOiMAGE Medical Technologies Corporation, Shanghai, China

**Keywords:** lumbar spine, forward–backward bending, *in vivo* kinematics, soft tissue artifacts, dual fluoroscopy

## Abstract

Traditional optical motion capture (OMC) with retroreflective markers is commonly used to measure joint kinematics but was also reported with unavoidable soft tissue artifacts (STAs) when quantifying the motion of the spine. Additionally, the patterns of the STA on the lumbar spine remain unclear. This study aimed to 1) quantify the *in vivo* STAs of the human lower back in three-dimensional directions during weight-bearing forward–backward bending and 2) determine the effects of the STAs on the calculated flexion angles between the upper and lower lumbar spines and adjacent vertebrae by comparing the skin marker (SM)- and virtual bone marker (VM)-based measurements. Six healthy volunteers were imaged using a biplanar radiographic system, and thirteen skin markers were mounted on every volunteer’s lower back while performing weight-bearing forward–backward bending. The STAs in the anterior/posterior (AP), medial/lateral (ML), and proximal/distal (PD) directions were investigated. The flexion angles between the upper and lower lumbar segments and adjacent intervertebral segments (L2–L5) throughout the cycle were calculated. For all the participants, STAs continuously increased in the AP direction and exhibited a reciprocal trend in the PD direction. During flexion, the STA at the lower lumbar region (L4–L5: 13.5 ± 6.5 mm) was significantly higher than that at the upper lumbar (L1–L3: 4.0 ± 1.5 mm) in the PD direction (*p* < 0.01). During extension, the lower lumbar (L4–L5: 2.7 ± 0.7 mm) exhibited significantly less STAs than that exhibited by the upper lumbar region (L1–L3: 6.1 ± 3.3 mm) (*p* < 0.05). The STA at the spinous process was significantly lower than that on both sides in the AP direction (*p* < 0.05). The present results on STAs, based on dual fluoroscopic measurements in healthy adult subjects, presented an anatomical direction, marker location, and anatomic segment dependency, which might help describe and quantify STAs for the lumbar spine kinematics and thus help develop location- and direction-specific weighting factors for use in global optimization algorithms aimed at minimizing the effects of STAs on the calculation of lumbar joint kinematics in the future.

## 1 Introduction

Low back pain (LBP) is one of the most common musculoskeletal disorders and a leading cause of disability, globally creating a substantial personal, community, and financial burden (Airaksinen et al., 2006; Delitto et al., 2012; Itz et al., 2013; Maher et al., 2017). Approximately 85%–95% of LBP cases have no identifiable cause or pathology and are, therefore, classified as non-specific LBP (NS-LBP). Hoy et al. (2012), Maher et al. (2017), and Wallbott (1989) characterized the way patients with NS-LBP perform daily activities and defined movement quality (MQ) as “the way in which human movement is executed with respect to the dimensions of time and space.” Observation, analysis, and the influence of whole-body movements on the MQ are the key elements of LBP management (Laird et al., 2012; Hodges et al., 2013).

The common final manifestation of LBP is a change in spine kinematics (Liguori and ACSM, 2021). Accurately quantifying three-dimensional (3D) joint kinematics during *in vivo* lumbar motion is essential for a detailed understanding of joint function, investigating pathologies, and assessing the success of therapies (Aiyangar et al., 2014). 3D motion analysis, such as optical motion capture (OMC), using skin markers is the most common method for measuring spine joint kinematics *in vivo* (Wang et al., 2016; Huang et al., 2021). Unfortunately, when skin-marker trajectories are used in human motion analysis, the skin-mounted markers move over the underlying bone, generating the so-called soft tissue artifact (STA), which makes the estimation of the instantaneous skeletal pose difficult (Leardini et al., 2005). Motions of soft tissue covering the spine may cause large errors (Mo¨rl and Blickhan, 2006). STA represents one of the main limitations in obtaining accurate and reliable skeletal kinematics from motion capture (Cereatti et al., 2017).

Many previous studies have demonstrated that the extent of STAs, caused by a combination of skin stretching and sliding, muscle contraction, gravity, and inertia, is unique to each specific body segment (Akbarshahi et al., 2010), the physical characteristics of individuals (Holden et al., 1997), marker locations (Schwartz et al., 2004), and the nature of the performed movement task (Fuller et al., 1997). Several studies have validated the STAs of skin markers on different body regions (Wang et al., 2016; Camomilla and Bonci, 2020; Metcalf et al., 2020). However, validation studies for lumbar spine segments are rare (Zemp et al., 2014, Mo¨rl and Blickhan, 2006). Moreover, most studies investigated lumbar STAs in a seated upright position (Mo¨rl and Blickhan, 2006) or static sitting position (Zemp et al., 2014), and a few have been validated against global spinal shape.

It is also important to quantify the propagation of STAs to estimate lumbar joint kinematics. Various studies have used intrusive techniques to quantify STAs; for example, intra-cortical pins (Fuller et al., 1997; Benoit et al., 2006; Dal Maso et al., 2016), external fixators (Cappozzo et al., 1996), and percutaneous skeletal trackers (Manal et al., 2000) have been used to quantify joint motion *in vivo*. Unfortunately, these devices restrict the movement of the participant and alter the normal, unimpeded sliding of the soft tissues relative to the underlying bone. Moreover, these techniques are invasive; therefore, their use is limited. To overcome these problems, non-invasive methods, such as magnetic resonance imaging (MRI) (Sangeux et al., 2006; Zemp et al., 2014) and X-ray fluoroscopy (Garling et al., 2007), have been used to quantify joint motion *in vivo*. However, the methods proposed in these studies have several limitations: 1) they capture several terminal static poses in different directions rather than measuring a continuous dynamic motion; 2) they use a skin marker set and compare the measurements collected from 2D images to quantify STAs at the lower back; and 3) they investigate only a single motor task (Garling et al., 2007; Zemp et al., 2014). The dual fluoroscopy imaging system (DFIS) has been recognized as an accurate and validated *in vivo* evaluation technique for determining vertebra translations and orientations with the accuracy of 0.3 mm and 0.70° (Wang et al., 2008). Recent studies have demonstrated that the motion tracking technique based on dual fluoroscopic imaging is a reliable method for determining intervertebral motion and adjacent segment kinematics in various functional spine motions (Wawrose et al., 2020; Zhou et al., 2020). Therefore, in this study, we used a DFIS combined with a validated 3D-to-2D registration technique (Wang et al., 2020; Zhou et al., 2021) to quantify *in vivo* intervertebral and upper-lower lumbar segment kinematics in asymptomatic subjects during forward–backward bending.

The present study aimed to describe patterns and magnitudes of STAs *in vivo* in three different directions on the full region of the human lumbar spine during weight-bearing forward–backward bending. Vertebral movement through the cycle was measured using the DFIS. It also aimed to determine the effects of STAs on the calculated flexion angles between the upper and lower lumbar spine and adjacent intervertebral levels for L2–L5 by comparing the skin marker (SM)- and virtual bone marker (VM)-based measurements. We hypothesized that the *in vivo* patterns of skin markers in the lumbar region would be direction- and segment-dependent patterns and that STAs would significantly affect the kinematic variables in human spinal motion investigation.

## 2 Materials and methods

### 2.1 Participant preparation

The present study was conducted with the approval of the institutional review board at Tongji Hospital, Shanghai, China (Protocol Number: 2021-011-SK), and followed the guidelines of the Helsinki Declaration (2013) (World Medical Association, 2013). The recruitment was conducted in the personal and workplace environment of the investigators. Ten asymptomatic adults without prior diagnosed spinal disorders were recruited. All the participants provided written informed consent before any personal or health-related data were collected. The inclusion criteria were as follows: age between 18 and 75 years, ability to perform the required functional tasks, and sufficient understanding of Chinese. Individuals were excluded in the case of any history of LBP in the past 6 months; injuries or operations on the spine, hip, knee, or ankle; and any comorbidities or circumstances (e.g., pregnancy) that could limit the forward–backward bending capabilities. Additionally, yoga practitioners, dancers, gymnasts, and those who had physical therapy within the last three months were ineligible owing to potential bias toward bending activities.

All the participants underwent a lumbar spine computed tomography (CT) scan (United Imaging, uCT760, voltage 120 kV, resolution 512 × 512, layer spacing 1.0 mm) in a supine, relaxed position. The CT images were imported into the 3D visualization and modeling software, Amira 6.7 (Thermo Fisher Scientific, Rockford, IL, United States), to reconstruct 3D bone models of the lumbar spine. All images were processed using Digital Imaging and Communications in Medicine (DICOM) and bitmap file formats. Four participants were excluded because of poor biplane image quality of the lumbar spine due to abdominal obesity and difficulties in later registration. Eventually, six pain-free female adults (age: 26.17 ± 3.55 years, body mass index (BMI): 19.76 ± 1.36 kg/m^2^) were included in this study.

### 2.2 Experiment procedure

**FIGURE 1:**
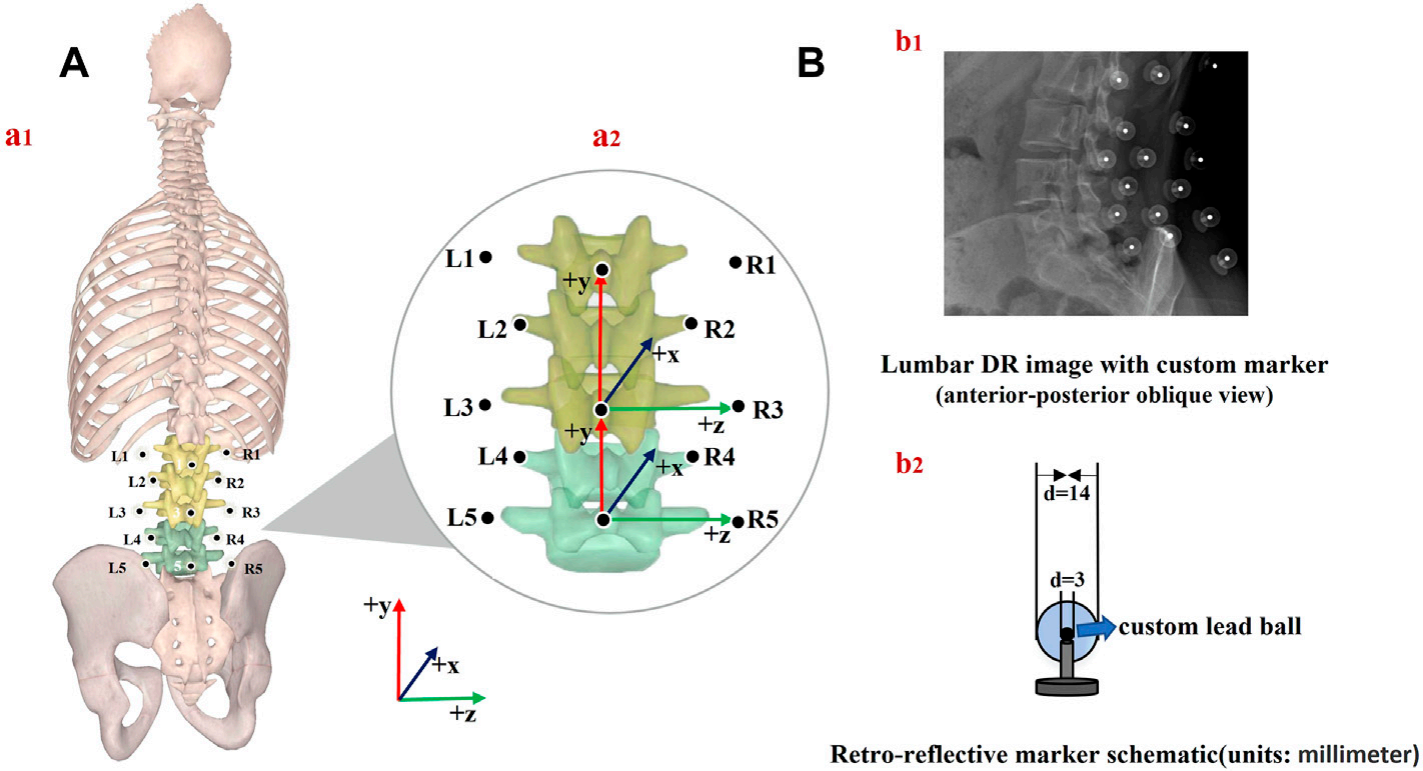
**(A)** Schematic of marker placement and spine anatomical frames of reference (a1); joint coordinate system axes of rotation for the upper and lower lumbar segment considered (a2)**, (B)** lumbar DR image (b1) and retroreflective marker schematic (b2).

A trained physiotherapist (7 years of experience in motion analysis) equipped all the participants through manual palpation with the subject standing upright. Based on our lumbar marker set (Gombatto et al., 2017; Papi et al., 2019), 13 custom reflective spherical markers (diameter: 14 mm) with a lead core (diameter: 3 mm) were used. The central marker was always positioned on the L1, L3, and L5 spinous processes (Markers 1, 3, and 5), which were identified following the palpation guidelines (Field and Hutchinson, 2012). Six lateral markers were placed 5 cm on either side from the spinous process of the first (Markers L1 and R1), third (Markers L3 and R3), and fifth (Markers L5 and R5) lumbar vertebrae separately. Four markers were attached 2.5 cm to each side of the spinous process of the second (Markers L2 and R2) and fourth (Markers L4 and R4) lumbar vertebrae (Papi et al., 2019). The primary goal of this marker set (Table1) was to constrain the lumbar segment (L1–L5) so that the flexion angles between the upper and lower lumbar spine and adjacent vertebrae could be obtained *via* kinematic calculation (Figure 1).

**TABLE 1:** Marker placement and abbreviations.

Abbreviations	Marker placement description
L5	5.0 cm left lateral back on the fifth lumbar vertebrae
5	Spinous processes of the fifth lumbar process
R5	5.0 cm right lateral back on the fifth lumbar vertebrae
L4	2.5 cm left lateral back on the fourth lumbar vertebrae
R4	2.5 cm right lateral back on the fourth lumbar vertebrae
L3	5.0 cm left lateral back on the third lumbar vertebrae
3	Spinous processes of the third lumbar process
R3	5.0 cm right lateral back on the third lumbar vertebrae
L2	2.5 cm left lateral back on the second lumbar vertebrae
R2	2.5 cm right lateral back on the second lumbar vertebrae
L1	5.0 cm left lateral back on the first lumbar vertebrae
1	Spinous processes of the first lumbar process
R1	5.0 cm right lateral back on the first lumbar vertebrae

**FIGURE 2:**
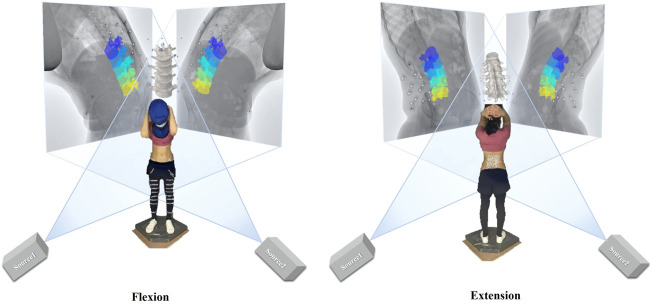
Experimental setup of the dual fluoroscopic system for capturing lumbar spine and marker positions *in vivo*; virtual reproduction of the dual fluoroscopic system and vertebral positions.

Quasi-static dual-digital radiography (DR) images (TAOiMAGE, Shanghai, China; image resolution 2804 × 2804 pixels) were initially captured during a static standing trial with the subject’s feet shoulder-width apart for two seconds. Next, each participant performed weight-bearing forward–backward bending from maximum extension to approximately 45° of flexion (measured using a protractor) to keep the participants within view of the system (Figure 2). In order to reduce the overlapping of skin markers and various internal bony structures in dual fluoroscopic lumbar spine images and enhance the efficiency and accuracy of registration and recognition, the participants stood in an oblique position of 45°. Before the formal collection, we pasted marked lines at different heights of the participants’ lower limbs, instructed the participants to place their palms in front of their lower limbs, and moved down to the designated marking line in an orderly manner. While reaching the designated marked position, they were asked to cross their hands behind their heads and keep still. Participants were trained to keep both knees straight throughout the process. After the preparation above, anteroposterior and lateral biplanar images of the lumbar vertebrae were recorded synchronously at different bending angles. Participants practiced the activities before the actual testing to get familiar with the tasks. The testing was repeated if the participants violated the task instructions, resulting in non-valid trials. During the experiment, all the participants were required to wear lead protective clothing in non-collection areas to reduce unnecessary radiation. During testing, the participant was exposed to approximately seven pairs of fluoroscopic projections. The entire image collection process took around 10 min to complete. According to the product manual provided by the manufacturer, the effective dose under our typical testing conditions (78 Kv, 40 mA) is 0.16 mSv for each paired image of the lumbar or less than 1.20 mSv per test, approximately 6% of the average dose limit for occupational exposure in a year.

### 2.3 *In vivo* kinematics measurements

Two multi-segmental kinematic models of the lumbar spine were used to derive lumbar spine kinematics based on virtual bone and skin markers. At each vertebral level and lumbar segment, anatomical coordinate systems were defined based on the recommendations of the International Society of Biomechanics (Wu et al., 2002).

1) At each vertebral level, the origin was in the middle of the upper and lower endplates. The *y*-axis (proximal/distal, PD) was defined as the line passing through the centers of the upper and lower vertebral endplates. The *z*-axis (medial/lateral, ML) was defined as the line parallel to a line joining similar landmarks on the bases of the right and left pedicles, pointing to the right. The *x*-axis (anterior/posterior, AP) was perpendicular to the *y*- and *z*-axes and pointed anteriorly.
2) The upper lumbar segment was defined by its origin at L3, a vertical axis from the spinous process of L3 to L1 (PD, +y), an AP axis (+x) orthogonal to the plane formed by the PD axis, the line connecting the left and right transverse processes of L3, and a horizontal axis (ML, +z) cross-product of AP and PD axes. The lower lumbar segment was defined by its origin at L5, a vertical axis from L5 to L3 (PD, +y), an AP axis orthogonal to the plane formed by the PD axis, the line connecting the left and right transverse processes of L5, and a horizontal axis cross-product of the AP and PD axes (Gombatto et al., 2017) [Figure 1A (a2)].

### 2.4 Data processing and analysis

The STA-free trajectories of the markers (i.e., “virtual bone markers”) were derived from the kinematics of the bones. 2D DR images and 3D models were imported into a registration program (MATLAB, R2018a MathWorks, Natick, MA, United States). The 3D positions of each lumbar vertebra and marker were adjusted until they matched the corresponding outlines on the DR images. The 3D positions of the skin markers relative to the underlying vertebra in each frame were defined. This technique was validated by the roentgen stereophotogrammetric analysis technique as the gold standard for achieving submillimeter accuracy in our preliminary study (Li et al., 2009). Bone landmark positions from CT measurements were transformed into the biplanar DR coordinate system and combined with a skin marker to determine the flexion angles of the upper lumbar relative to the lower lumbar segment. In order to evaluate the higher derivatives of the motion parameter based on the two systems, flexion angles between the adjacent lumbar vertebra (L2–L3, L3–L4, and L4–L5) were also examined. Lumbar joint angles were calculated using the relevant segment poses following a z-x-y cardanic rotation sequence (Robertson et al., 2004; Lees et al., 2010), which corresponded to flexion+/extension−, right bend+/left bend−, and right twist+/left twist−. The effects of STAs on lumbar kinematics were quantified throughout the motion using the differences between the SM-determined and VM-determined kinematics.

Each local coordinate system was located at the center of gravity of the respective vertebral bodies. The global coordinates of the markers were converted into the corresponding local vertebral coordinate system. STA was calculated by subtracting the displacement of the skin marker under the coordinate system of each vertebra from the corresponding marker in the static standing position. The overall flexion angle of the lumbar spine was described as the angle between the fitting line through the center of all vertebrae in each frame and the static standing position. The STA at each tested flexion angle of each subject was normalized to one bending cycle, from full extension (0% of normalized cycle) to maximal flexion (100% of normalized cycle), through data interpolation. The flexion angle ranged from −15° to 45° with an interval of 7.5°. The mean values and range of the STAs in the AP, ML, and PD directions among all the participants were investigated. The mean STAs at the lower lumbar (Markers R5, 5, L5, R4, and L4) and upper lumbar regions (Markers L3, 3, R3, L2, R2, L1, 1, and R1) among all the participants during extension and flexion were calculated. Meanwhile, the STAs at the spinous processes (Markers 1, 3, and 5) and both sides (Markers L1, R1, L2, R2, L3, R3, L4, R4, L5, and R5) were calculated.

### 2.5 Statistical analysis

Continuous variables are presented as means and standard errors. Normal distribution was verified using the Shapiro Wilk test and Q-Q-plot inspection for discrete parameters. A paired *t*-test with a significance level of 0.05 was used to compare 1) the STAs at the lower and upper lumbar regions, 2) the STAs at the spinous processes and both sides, and 3) the SM- and VM-determined flexion angles among all the participants at each tested frame. The correlation between the flexion angles based on the two measurements throughout the entire cycle was estimated using the Spearman correlation coefficient.

## 3 Results

### 3.1 Lumbar soft tissue artifact patterns and magnitudes in three directions during forward–backward bending

#### 3.1.1 Anterior–posterior direction

**FIGURE 3:**
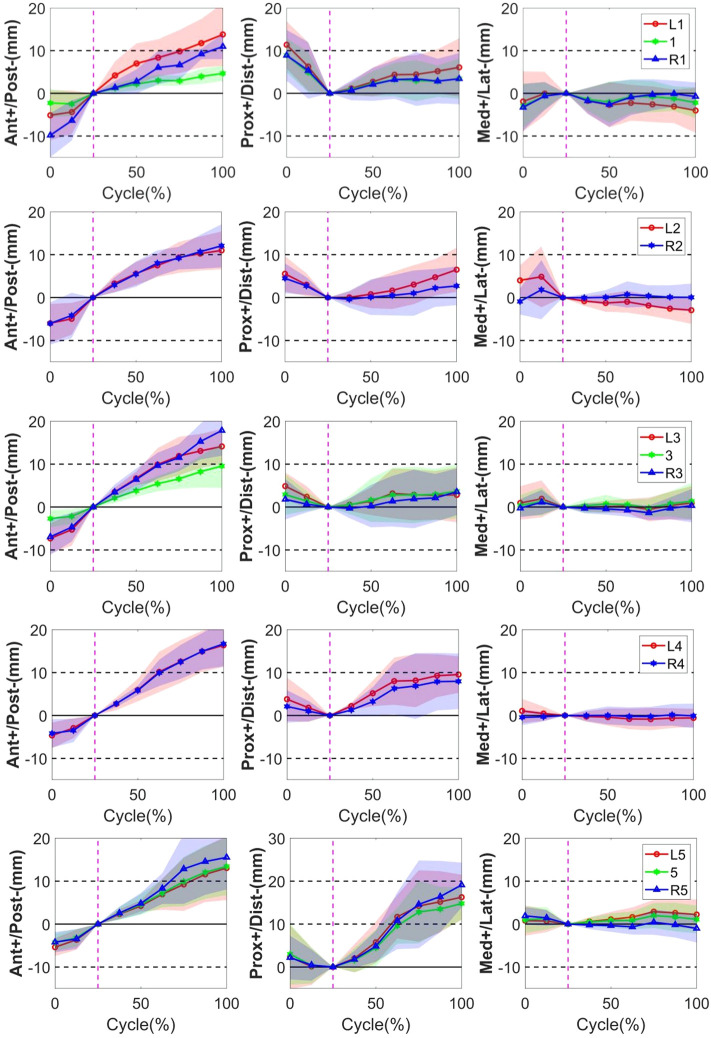
STA of the thirteen skin markers from L1 to L5 level during the bending cycle (0%, 25%, and 100% represent extension, neutral, and flexion, respectively). The solid line represents the mean STA for all the subjects, and the shaded-area error bands represent the standard deviation of the STA. The vertical dotted line represents the neutral position.

The quantitative plots of STAs during the bending cycle demonstrated that the amount of movement of skin markers relative to the underlying bi-planar DR-measured bone STAs at each lumbar level showed a similar increasing trend from full extension to flexion in the AP direction (Figure 3). All the markers were anteriorly shifted during the entire cycle. The smallest STA through the bending cycle occurred at marker spinous processes of L1, ranging from −2.3 ± 3.3 mm at full extension to 4.7 ± 1.9 mm at maximal flexion. The largest STAs occurred at the marker right side of the spinous process of L3, ranging from −6.9 ± 4.2 mm to 17.8 ± 5.9 mm (Table 2).

**TABLE 2:** Direction-related (AP, PD, and ML) mean marker artifact (mean) with their standard deviations (SDs) of all the lumbar skin markers in the flexed and extended positions.

Marker	AP (mm)		PD (mm)		ML (mm)	
	Ext. (mean ± SD)	Flex. (mean ± SD)	Ext. (mean ± SD)	Flex. (mean ± SD)	Ext. (mean ± SD)	Flex. (mean ± SD)
L5	−5.4 ± 2.1	13.0 ± 7.5	2.3 ± 7.5	16.3 ± 5.2	0.8 ± 3.5	2.2 ± 3.5
5	−4.3 ± 2.0	13.4 ± 6.7	3.0 ± 7.1	14.8 ± 3.8	1.0 ± 3.1	1.1 ± 3.2
R5	−4.2 ± 2.4	15.5 ± 7.5	2.2 ± 5.1	19.1 ± 5.2	1.9 ± 2.0	−1.0 ± 3.3
L4	−4.7 ± 3.0	16.4 ± 5.1	3.8 ± 5.1	9.5 ± 4.3	1.1 ± 2.9	−0.6 ± 2.1
R4	−4.2 ± 3.3	16.7 ± 5.2	2.1 ± 3.8	8.0 ± 6.5	−0.4 ± 1.8	−0.1 ± 2.9
L3	−7.3 ± 3.5	14.1 ± 4.1	4.9 ± 3.2	2.8 ± 6.4	0.9 ± 3.9	0.9 ± 3.9
3	−2.7 ± 2.0	9.6 ± 5.1	2.9 ± 4.7	3.7 ± 6.3	0.4 ± 2.4	1.3 ± 4.3
R3	−6.9 ± 4.2	17.8 ± 5.9	1.8 ± 4.7	3.6 ± 5.4	−0.3 ± 2.1	0.3 ± 3.9
L2	−6.0 ± 4.5	11.0 ± 4.4	5.5 ± 4.1	6.5 ± 5.2	4.0 ± 3.6	−2.9 ± 3.2
R2	−6.0 ± 4.9	12.1 ± 5.0	4.5 ± 3.5	2.7 ± 4.5	−0.9 ± 3.0	0.6 ± 3.2
L1	−5.1 ± 5.9	13.8 ± 7.3	11.4 ± 5.6	6.1 ± 6.9	−1.9 ± 7.1	−4.0 ± 5.3
1	−2.3 ± 3.3	4.7 ± 1.9	8.9 ± 4.0	3.4 ± 4.7	−2.9 ± 5.2	−2.2 ± 3.6
R1	−9.8 ± 5.2	11.0 ± 2.9	8.9 ± 6.0	3.4 ± 6.0	−3.3 ± 5.5	−0.8 ± 3.4

AP, anterior/posterior; ML, medial/lateral; PD, proximal/distal; Flex., flexion; Ext., extension.

#### 3.1.2 Proximal–distal direction

In the PD direction, the STA exhibited an approximately reciprocal pattern during the lumbar extension and flexion (Figure 3). The STA underwent a sharp increase during flexion at the L5 level (Markers L5, 5, and R5 shifted 16.3 ± 5.2 mm, 14.8 ± 3.8 mm, and 19.1 ± 5.2 mm proximal from the static position to maximal flexion, respectively), followed by the L4 level (Markers L4 and R4 shifted 9.5 ± 4.3 mm and 8.0 ± 6.5 mm proximal from the static position to maximal flexion, respectively), and slightly increased at the L1-L3 level (maximal STA < 6.5 mm). At the extension phase, the STA in the PD direction slightly decreased at the L3–L5 level (maximal STA < 4.9 mm), and the greatest decrease occurred at the L1 level (Markers L1, 1, and R1 shifted 11.4 ± 5.6 mm, 8.9 ± 4.0 mm, and 8.9 ± 6.0 mm distal from full extension to the static position, respectively), followed by L2 (Markers L2 and R2 shifted 5.5 ± 4.1 mm and 4.5 ± 3.5 mm distal from full extension to the static position, respectively) (Table 2). The positions of the markers did not move either to the right or left during the bending motion.

#### 3.1.3 Medial–lateral direction

Throughout the bending cycle, the changing trend of the STA in the ML direction was stable relative to the AP and PD. In the extension phase, the largest STA occurred at Marker R1 (−3.3 ± 5.5 mm), and the smallest STA occurred at Marker R3 (−0.3 ± 2.1 mm). During flexion, the smallest STA occurred at Marker R4 (0.1 ± 2.9 mm), and the largest STA occurred at Marker L1 (−4.0 ± 5.3 mm) (Table 2).

In conclusion, as the gross spinal angle increased, the STA became prominent in the AP and PD directions. Specifically, the skin markers moved from posterior to anterior with respect to the underlying vertebra through the bending cycle in AP, whereas, in the PD direction, it shifted from proximal to distal during extension and from distal to proximal during flexion (Figure 3).

### 3.2 Quantification and characteristics of lumbar soft tissue artifact in upper and lower lumbar regions during forward–backward bending

The results showed that the range of STAs during flexion was 13.0 ± 3.4, 7.7 ± 5.6, and −0.8 ± 1.8 mm in the AP, PD, and ML directions, respectively, and −5.2 ± 2.0, 4.8 ± 3.1, and −0.7 ± 2.0 mm in the AP, PD, ML directions, respectively, during extension. During flexion, the STA at the lower lumbar (L4, L5: 13.5 ± 6.5 mm) was significantly higher than that at the upper lumbar regions (L1–L3: 4.0 ± 1.5 mm) in the PD direction (*p* < 0.01). During extension, the STA at the lower lumbar (L4–L5: 2.7 ± 0.7 mm) was significantly less than that at the upper lumbar region (L1–L3: 6.1 ± 3.3 mm) in the PD direction (*p* < 0.05). The STA at the spinous process was significantly lower than that on both sides in the AP direction during extension and flexion (*p* = 0.02) (Table 3).

**TABLE 3:** Segment-related (upper lumbar, lower lumbar) mean marker artifact (mean) with their standard deviations (SD) of the lumbar skin markers in the flexed and extended positions and the 95% confidence interval (95% CI) of difference.

Segment	Flex. (mm)			Ext. (mm)		
	AP (mean ± SD)	PD (mean ± SD)	ML (mean ± SD)	AP (mean ± SD)	PD (mean ± SD)	ML (mean ± SD)
Upper lumbar	11.8 ± 3.8	4.0 ± 1.5	−0.9 ± 2.0	−5.8 ± 2.5	6.1 ± 3.3	−0.5 ± 2.4
Low lumbar	15.0 ± 1.7	13.5 ± 6.5∗	0.3 ± 1.3	−4.6 ± 0.5	2.7 ± 0.7∗	0.9 ± 0.8
*p*-value	0.105	0.009∗	0.268	0.309	0.023∗	0.239
95%CI of difference	(−7.3,0.8)	(−15.2,3.8)	(−3.4,1.0)	(−3.7,1.3)	(0.6,6.2)	(−3.8,1.1)

AP, anterior/posterior; ML, medial/lateral; PD, proximal/distal; Flex., flexion; Ext., extension.

∗Significant differences with upper lumbar (*p* < 0.05).

### 3.3 Effects of soft tissue artifact on the calculation of flexion angles between the upper and lower lumbar spine and adjacent intervertebral levels

**FIGURE 4:**
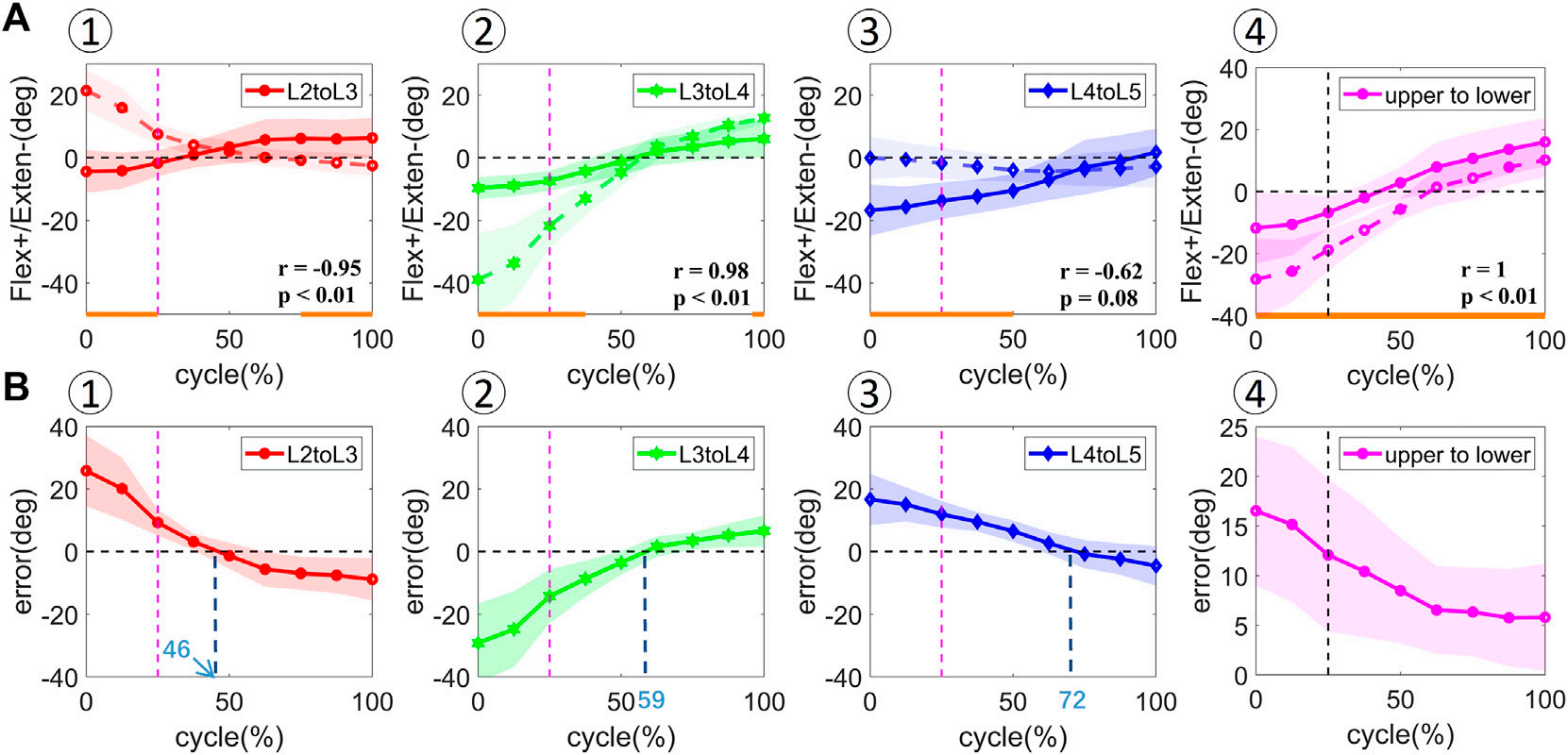
[**(A)** ①–④] *In vivo* kinematics measured by two measurements at adjacent vertebral levels (L2–L3, L3–L4, L4–L5) and upper and lower lumbar levels (upper to lower). The solid line represents the mean values for all the subjects measured by DR. The dotted line represents the mean values for all the subjects measured by skin markers. Statistical significance between the two measurements is marked by the solid orange line along the *x*-axis of top-row graphs. [**(B)** ①–④] Rotational error at adjacent vertebral levels (L2–L3, L3–L4, L4–L5) and upper and lower lumbar levels (upper to lower). The shaded area represents the standard deviation of the values.

Significant effects of STAs on the calculated vertebral flexion angles were observed. VM measurement demonstrated a consistent increase in intervertebral flexion with overall body flexion, with an intervertebral range of motion (ROM) of 2.70 ± 4.23°, 2.30 ± 2.74°, and 3.06 ± 3.95° during extension and 8.62 ± 5.78°, 13.57 ± 3.36°, and 15.39 ± 2.61° during flexion for L2–L3, L3–L4, and L4–L5 levels, respectively. The overall patterns of joint angles measured by SM over the bending cycle were opposite to VM for the L2–L3 (*r* = −0.95, *p* < 0.01) and L4–L5 levels (*r* = −0.62, *p* = 0.08), but similar for the L3–L4 level (*r* = 0.98, *p* < 0.01) (Figure 4). However, the amplitude of variation of the flexion angle at the L3–L4 level was overestimated by SM measurements, with L3 more extended during the 0%–50% cycle and more flexed during the 50%–100% cycle. Significant differences in the joint angles among all the participants occurred during the 0%–25%, 75%–100%; 0%–37.5%, 100%, and 0%–50% cycles for L2–L3, L3–L4, and L4–L5 levels, respectively (*p* < 0.05). The zero kinematic errors of L2/L3, L3/L4, and L4/L5 under the two measurement methods are at 46%, 59%, and 72%, respectively (Figure 4), indicating that the skin movement characteristics of the upper and lower lumbar segments were different. These discrepancies can be explained by the skin deformation mechanism caused by complex lumbar muscle deformation. Second, it is speculated that the kinematic error of zero for the two measurements at 50% of the bending cycle occurs near the L3 level, which may be related to its anatomical and biomechanical characteristics because L3 is the apex of lumbar lordosis and the center of lumbar motion.

The flexion angles between the upper and lower lumbar levels from full extension to maximal flexion demonstrated a similar increasing trend in SM and VM measurements (Figure 4). The VM- and SM-derived flexion angles were 4.99 ± 6.57° and 9.44 ± 11.84° during extension and 23.53 ± 4.66° and 29.00 ± 7.87° during flexion, respectively. A significant difference in the flexion angle was observed among all the participants during the entire cycle (*p* < 0.05). A linear relationship was found between the flexion angles during the bending cycle and both measurements (*r* = 1, *p* < 0.01). The error in the measured flexion angle decreased from the extension to the flexion phase.

## 4 Discussion

The most important finding of the present study is that the extent of STAs in the lumbar spine differed by anatomical direction, marker location, lumbar segment, and bending phases when the participants performed forward–backward bending under the weight-bearing condition. Specifically, the STA was prominent in the AP and PD directions. As the flexion angle increased, the STA continuously increased in the AP direction and exhibited a reciprocal trend in the PD direction during extension and flexion. The STA in the lower lumbar region was significantly smaller than that in the upper lumbar region during extension and larger during flexion in the PD direction. The STA at the spinous process was significantly smaller than that on either side. These findings were consistent with those of the previous studies that described the factors that influence the STA extent (Fuller et al., 1997; Holden et al., 1997; Schwartz et al., 2004). These specific patterns and magnitudes of STAs in the lumbar spine might help clinicians and physical therapists assess kinematic errors and devise methods to minimize its effects on the calculation of lumbar joint kinematics.

STA is particularly evident in the analysis of the lumbar spine because the markers are close to each other, and small relative movements among them may lead to large errors in the assessment of vertebral position and orientation (Ciavarro et al., 2006). Previous studies showed that some general features might be identified. For instance, for tasks performed by non-obese volunteers and motor tasks that do not involve high accelerations or impacts, the largest proportion of the STA-affecting markers located on a given body segment is closely correlated to the relevant joint movement (Cappozzo et al., 1996; Bonci et al., 2014; Camomilla et al., 2015). Evidence showed that forward bending (Corkery et al., 2014; van Wingerden et al., 2008), lifting (Hemming et al., 2018; Pranata et al., 2018), and walking (Henchoz et al., 2015; Lamoth et al., 2006) are key activities in assessing subjects with NS-LBP, also corresponding to the most frequently reported treatment goals of patients with LBP on the Patient-Specific Functional Scale (Stratford, 1995). In this study, we chose forward–backward bending, which is relatively simple in execution and acquisition, as the daily activity for evaluating MQ in healthy participants. To ensure the accuracy and validity of the registration and final quantification data, we finally selected six participants with a relatively low BMI and good image quality.

To the best of our knowledge, only a few studies have reported STA results in the spinous process (Mo¨rl and Blickhan, 2006; Zemp et al., 2014). They showed that the largest STA was 27.4 mm for marker spinous process L5 in the flexed position, and the largest absolute value of STA was 18.3 mm for marker spinous process L3 in the extended position (Zemp et al., 2014). It is higher than our results for the corresponding marker location and task position (L3: 7.6 mm, L5: 20.1 mm). This may be because our experiment was performed under the weight-bearing condition, which is different from those in previous studies using magnetic resonance imaging MRI or CT machines, which limit the positions of the subject to only passive posture (Mo¨rl and Blickhan, 2006; Zemp et al., 2014). In fact, increasing the load significantly affects the movement of the lumbar joints (Wen et al., 2022), and flexion of the spine under active conditions is the most common mechanical cause of lumbar injuries (Shahbazi Moheb Seraj et al., 2018). During extension, the STA in the lower lumbar region was significantly smaller than that in the upper lumbar region in the PD direction, whereas, in the flexion phase, the STA gradually increased from proximal to distal. The maximum STA reached approximately 20 mm at the L5 level, followed by approximately 10 mm at the L4 level, and fell below 10 mm from L1–L3. A high degree of lumbar STA variability was observed during lumbar extension and flexion. The characteristics of STAs at each specific lumbar level may help quantitatively describe the lumbar segment-related kinematic errors caused by STAs, thus providing a better understanding of the normal and pathological biomechanics of the lumbar spine during functional activities.

Various marker placement methods have already been used to track the movement of the human lumbar spine, which is mainly separated into upper and lower segments, to study the kinematic characteristics of LBP (Gombatto et al., 2015; Hemming et al., 2018; Papi et al., 2019). Our study is the first to investigate the *in vivo* 3D STA of markers on either side of the spinous process. The overall direction of the marker shift was similar to that reported in a previous study (Zemp et al., 2014). We demonstrated that the 3D STA at the spinous process was significantly smaller than that on either side, which means that markers at anatomical landmarks would be least affected by lumbar motion. This is consistent with not only the STA’s markers’ location-related characteristics but also the theoretical conjecture. Because the marker at the spinous process is the closest to the osseous structure of the internal spinous process, there are relatively few soft tissues, such as muscle and fat, between the two, which ultimately results in the least amount of surface-skin deformation caused by internal-muscle deformation.

The present study demonstrated a linear correlation between the flexion angle measured using skin markers and dual DR focused on the upper and lower lumbar segments. This result supports previous findings that the marker set is reliable in assessing lumbar kinematics regardless of the STAs (Seerden et al., 2016). On the contrary, intervertebral rotations measured by the skin markers in our study were far from the intrinsic values measured by fluoroscopy for the L2–L3 and L4–L5 levels. Although a high correlation between the flexion angles at the L3–L4 level *(r* = 0.98, *p* < 0.01) derived by the two methods was found, the irregular STA of markers on different lumbar levels in the AP direction together led to a significant overestimation of the flexion angles. The consistency of marker positions and vertebral motion at the L3–L4 level was aligned with some previous studies (Mo¨rl and Blickhan, 2006; Hashemirad et al., 2013); however, the errors should be considered when performing marker-based motion tracking on the lumbar spine. Moreover, given that the overall distribution of the STAs in the lumbar area is lacking, the effectiveness of our markers in estimating the joint angles needs further verification.

This study had several limitations. First, only young women with relatively low BMI were included in the experiment. This experiment was designed to maintain high image quality for accurate *in vivo* vertebral-motion tracking with thinner fat on the back of the participants. However, large individual differences still exist because of the heterogeneity of each person. While the STA and its effects on spinal kinematics may be affected by the subject’s BMI, age, and sex (Tsai et al., 2011), participants of a larger age and male sex could be involved in the future. Second, to improve the accuracy of STAs and reduce unnecessary radiation, we focused on the flexion-extension direction for acquisition and analysis; therefore, the distribution characteristics and quantitative results of lumbar spine STAs in other degrees of freedom were lacking. However, learning the displacement of the marker set in the full region of the lumbar forward–backward bending could provide a useful reference for estimating 6DOF lumbar motions using SMs. Third, the quasi-static images captured in the current study presented the STA during different bending phases but still could not represent the instantaneous dynamic positions of the marker relative to the vertebra. However, “quasi-static” indicates a dynamic effect, and quasi-static load indicates that the load is applied slowly such that the structure deforms significantly slowly (at a considerably low strain rate); therefore, the inertial force is significantly small and can be ignored. Last but not least, the experimental collection was carried out asynchronously under the motion capture and DFIS, and the errors in OMC measurement differ from the SM measured radiographically, which might limit the utility of this study in future research. However, the quantitative results and patterns of STAs on the lumbar spine might help develop factors for use in global optimization algorithms aimed at minimizing the effects of STAs on the building of specific lumbar kinematic models and calculation of lumbar joint kinematics in the future.

In summary, the extent of STAs in the lumbar spine differed with respect to anatomical direction, marker location, lumbar segment, and flexion phases when the participants performed forward–backward bending under the weight-bearing condition. The STA was prominent in the AP and PD directions during the forward–backward bending motion. As the flexion angle increased, the STA continuously increased in the AP direction and exhibited a reciprocal trend in the PD direction during extension and flexion. The STA in the lower lumbar region was significantly smaller than that in the upper lumbar region during extension and larger during flexion in the PD direction. Skin markers at the spinous process were least affected by the motion of the lumbar spine. The STA characteristics at different lumbar vertebral levels may help clinicians and physical therapists assess kinematic errors and devise methods to minimize its effects on the calculation of lumbar joint kinematics. Credible and valid lumbar spine kinematic results are important for future studies aiming to provide personalized clinical management for LBP.

## Data Availability

The raw data supporting the conclusions of this article will be made available by the authors without undue reservation.
